# ICEO, a biological ontology for representing and analyzing bacterial integrative and conjugative elements

**DOI:** 10.1038/s41597-021-01112-5

**Published:** 2022-01-20

**Authors:** Meng Liu, Jialin Liu, Guitian Liu, Hui Wang, Xiaoli Wang, Zixin Deng, Yongqun He, Hong-Yu Ou

**Affiliations:** 1 State Key Laboratory of Microbial Metabolism, Joint International Laboratory on Metabolic & Developmental Sciences, School of Life Sciences & Biotechnology, Shanghai Jiao Tong University, Shanghai, 200030 China; 2 Department of Critical Care Medicine, Ruijin Hospital, Shanghai Jiao Tong University School of Medicine, Shanghai, 200025 China; 3 State Key Laboratory of Pathogens and Biosecurity, Beijing Institute of Microbiology and Epidemiology, Beijing, 100071 China; 4 University of Michigan Medical School, Ann Arbor, MI 48109 USA

**Keywords:** Bacterial genetics, Gene ontology

## Abstract

Bacterial integrative and conjugative elements (ICEs) are highly modular mobile genetic elements critical to the horizontal transfer of antibiotic resistance and virulence factor genes. To better understand and analyze the ongoing increase of ICEs, we developed an Integrative and Conjugative Element Ontology (ICEO) to represent the gene components, functional modules, and other information of experimentally verified ICEs. ICEO is aligned with the upper-level Basic Formal Ontology and reuses existing reliable ontologies. There are 31,081 terms, including 26,814 classes from 14 ontologies and 4128 ICEO-specific classes, representing the information of 271 known experimentally verified ICEs from 235 bacterial strains in ICEO currently and 311 predicted ICEs of 272 completely sequenced *Klebsiella pneumoniae* strains. Three ICEO use cases were illustrated to investigate complex joins of ICEs and their harboring antibiotic resistance or virulence factor genes by using SPARQL or DL query. ICEO has been approved as an Open Biomedical Ontology library ontology. It may be dedicated to facilitating systematical ICE knowledge representation, integration, and computer-assisted queries.

## Introduction

Integrative and conjugative elements (ICEs), previously named conjugative transposons, are important bacterial mobile genetic elements (MGEs) and active contributors in horizontal gene transfer^1–4^. ICEs are usually integrated into bacterial chromosomes; once induced or activated, they can transmit between bacterial cells through the self-encoded functional conjugation machinery, such as the type IV secretion system (T4SS)^2,3^. Typically, ICEs have highly mosaic modular structures, including the recombination, conjugation, regulation, and accessory modules^5,6^. ICEs are more widespread in prokaryote genomes than conjugative plasmids and are thought to be the most prevalent self-transmissible conjugative elements^3,5^. Based on the dependence or independence of T4SS, ICEs can be categorized as T4SS-type ICEs and actinomycete ICEs (AICEs)^6^. T4SS-type ICEs are widely distributed both in Gram-negative and -positive bacteria, while AICEs have only been found in *Actinobacteria*.

ICEs confer hosts with many critical bacterial phenotypes and play a vital role in the process of bacterial adaptation and genome evolution, by carrying and disseminating cargo genes encoding for antibiotic resistance, heavy metal resistance, carbon-source utilization, antibiotic molecule (bacteriocin) synthesis, symbiosis, and pathogenesis and other adaptive phenotypes^3,4^. Virulence factor (VF) genes and acquired antibiotic resistance genes (ARGs) are commonly found in ICEs. And they may cause critical threats to human by disseminating between bacteria with the transfer of ICEs^7,8^. For example, *Klebsiella pneumoniae* is one of the most important Gram-negative human pathogens with multi-drug resistances^7^. ICE*Kp1* has been reported to be highly abundant in *K. pneumoniae*^9,10^, and carries the biosynthesis gene clusters of two known important virulence factors, yersiniabactin and colibactin^11^.

Information of thousands of experimentally validated or computationally predicted bacterial ICEs has been collected and stored in the freely accessible database ICEberg2^6^. The information archived includes the basic features, such as the size, GC content, host organism, and reference source, as well as the complex gene list and modular information about more than 1,000 ICEs. However, to make the best use of these collected data and the ongoing increase of ICE information, and to facilitate more effective and accurate identification and annotation of ICEs from single bacterial genomes and metagenomes, a knowledge base of bacterial ICEs in a machine-interpretable format is demanded^12^. The usage of structured ontology provides a feasible solution.

An ontology is a machine-interpretable controlled vocabulary of hierarchical and interconnected entities that emphasize the logical organization and representation of complex data and knowledge^13^. Structured ontologies have been used widely in biological/biomedical data and metadata standardization, integration, sharing, and analysis^14–17^. For example, one of the most successful and widely-used ontology, Gene Ontology (GO)^18^, which represents the information of cellular components, biological processes, and molecular functions, has been used as the standard to describe the functions of genes and gene products across different databases and to conduct various gene expression analyses. Various ontologies have been developed to support standard knowledge and data representation, integration, and computer-assisted analysis^15^. In 2007, a collective of ontology developers initiated the Open Biological and Biomedical Ontologies (OBO) Foundry and established a set of principles for the development of interoperable ontologies^13^. Only ontologies follow a rigorous and collaborative development process, meet the expectations and requirements, can be accepted into the OBO ontology library.

In this study, we report the development of an Ontology of the Integrative and Conjugative Element (ICEO). It is aimed to ontologically represent and integrate the ICE gene information and functional modules to support automatically computer-assisted reasoning and advanced analysis. The ICEO has logically represented and organized the information about the 271 experimentally verified ICEs from the ICEberg database. In addition, 311 *in silico* identified and manually curated ICEs from 272 *K. pneumoniae* strains were also included. ICEO was developed using state-of-the-art ontology engineering technologies^13,19^. A systematic analysis of the ICEO-represented knowledge base might allow us to generate new insights about these widely distributed integrative genetic elements.

## Results

### ICEO top-level design and ontology alignment

**Fig. 1:**
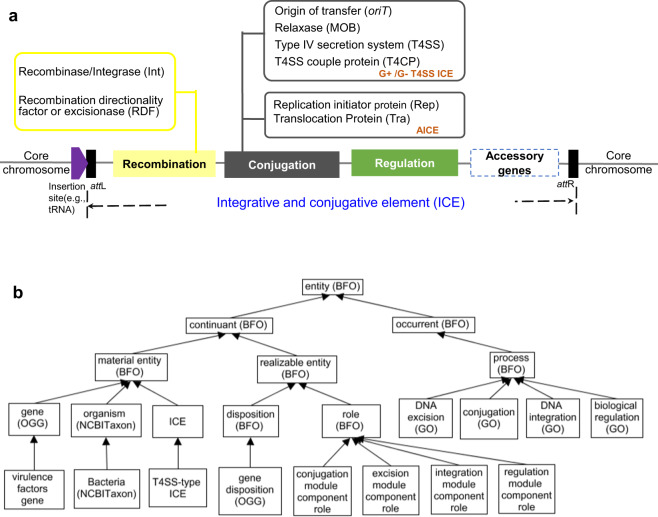
ICEO top-level design in alignment with the ICE functional modules. **(a)** Classical ICE conserved modules. ICEs typically contain three core modules: (i) a recombination (integration and excision) module, (ii) a conjugation module, and (iii) a regulation module. In addition, most ICEs possess conserved accessory regions. **(b)** ICEO top-level hierarchy. See details in the main text. Terms with ontology abbreviations inside parentheses are imported from external ontologies, while terms without an identified source are ICEO terms. Some intermediate terms such as those terms in between different layers are not shown to make the relations simple and clear. All the arrows indicate the ‘*is a*’ relation.

ICEO top-level design follows the ICE genetic functional modules. Figure 1a illustrates the genetic functional modules of the ICEs abundant in both Gram-positive and Gram-negative bacteria. A typical ICE includes three core modules functioning for recombination (integration and excision), conjugation, and regulation^2,3^. The recombination module refers to those genes and non-coding sequences responsible for the site-specific integration and excision of the elements from the host chromosomes. The conjugation module includes those genes and non-coding sequences involved in the conjugal process, such as genes encoding the relaxase and type IV secretion system (T4SS). The regulation module contains those genes and non-coding sequences contributing to the stabilization and maintenance. In addition to these three core modules, most ICEs carry cargo genes (also called accessory genes), such as the virulence factor (VF) genes and antibiotic resistance genes (ARGs).

Figure 1b represents the basic top-level ICEO hierarchical structure. Specifically, ICEO is aligned to the upper-level Basic Formal Ontology (BFO) 2.0 version^20^. BFO consists of ‘continuant’ and ‘occurrent’ branches. The ‘continuant’ branch stands for time-independent entities (*e.g*., material entity and their quality and roles), while the ‘occurrent’ branch represents time-related entities (*e.g*., process and time). BFO has been approved to be a top-level ontology standard by the International Standard Organization (https://www.iso.org/standard/74572.html). Since BFO has been used by over 250 ontologies, the alignment of ICEO with BFO facilitates the effective integration of ICEO with other existing ontologies. ICEO imports many related terms and relations from OBO library ontologies (Fig. 1b). The Ontology of Genes and Genomes (OGG)^21^ terms are imported to represent the genes of ICEs. NCBITaxon (a taxonomy ontology of NCBI organismal classification)^22^ (http://purl.obolibrary.org/obo/ncbitaxon) terms are imported to represent various ICE-containing organisms in the taxonomic organism hierarchy. Gene ontology (GO)^18^ terms are imported to represent the processes in the whole life cycle of ICEs.

Since ICE is essentially a genetic feature, we put our priority on the representation of the gene information rather than protein information. As detailed in the Methods section, ICEO applies an extensive gene ID assignments and label naming strategy by aligning to OGG*.* For example, the *ybtE* gene in *Klebsiella pneumoniae* strain NTUH-K2044 has a locus tag of *KP1_3592*. Accordingly, we assign this *ybtE* gene label as ‘KP1_3592(ybtE)’ and assign its gene ID as “OGG_KP1_3592” (Supplementary Figure S1).

### ICEO ontology design pattern

**Fig. 2:**
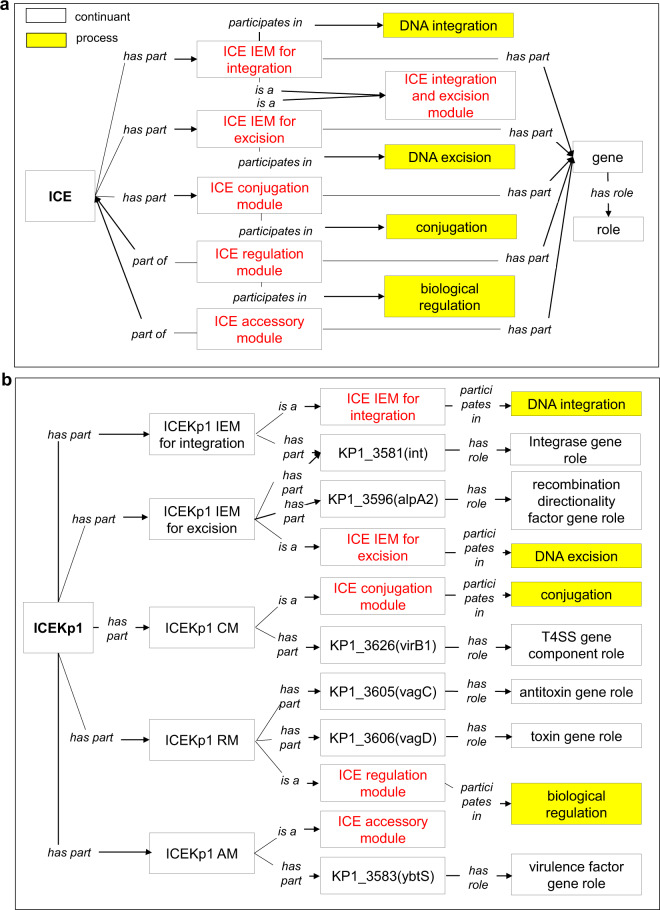
ICEO design pattern and example. **(a)** Generic ontology design pattern for relations among terms in ICEO. **(b)** An example of ICE*Kp1* representation using the ICEO design pattern. The knowledge about this ICE is obtained from the ICEberg database. All the relations are italicized. AM: Accessory module. CM: Conjugation module. IEM: Integration and excision module. RM: Regulation module. See main text for details.

Figure 2 illustrates the ICEO ontology design pattern to logically link different types of entities. The object property *‘participates in’* (RO_0000056) imported from Relation Ontology (RO)^23^ is used to express that an ICE functional module is involved in a biological process. Another RO object property *‘has role’* (RO_0000087) is also imported to express that a gene can serve as a specific role in the ICE system. The basic ICEO design pattern represents ICEs from the view of typical function modules (Fig. 1a). An ICE ‘*has part*’ integration, excision, conjugation, regulation, and accessory module components. Each of these components ‘*participates in*’ a specific ICE life process.

This general design pattern was applied to represent ICE*Kp1*, a virulence-associated ICE found in primary liver abscess-causing *K. pneumoniae* strain NTUH-K2044, the causative agent of primary liver abscess^11,24^. ICE*Kp1* contains all essential genes necessary to the whole life cycle, including the processes of excision, conjugation, regulation, and integration (Fig. 2b). The cargos of ICE*Kp1* include a cluster of genes responsible for the synthesis, regulation, and transport of siderophore yersiniabactin, conferring high virulence to the bacterium^11,24^.

Supplementary Figure S2 demonstrates how the different ICE components in the above ICE*Kp1* example are logically represented in the ICEO ontology. Each of the terms and relations is shown in the Protégé OWL editor. They are readable by humans and also interpretable by computers since these have been assigned specific Uniform Resource Identifiers (URIs). Computer interpretation is the basis of its future usage in various artificial intelligence applications. In addition, the demonstration of the well-documented ICEs, SXT(MO10) and Tn916, are shown in Supplementary Figures S3 and S4, respectively.

### ICEO Statistics

The latest release of ICEO (version 2.1) contains a total of 31,081 terms, including 30,942 classes, 53 object properties, and 80 annotation properties (Table 1). Among these 30,942 classes, 4,128 classes have ICEO_ namespace; the remaining terms were imported from various reliable OBO ontologies, such as BFO (19 classes), OGG (26,448 classes), NCBITaxon (321 classes), and GO (11 classes). There are 11 object properties and 21 annotation properties with the ICEO_ namespace. The other terms are also imported from OBO library ontologies, like RO (27 object properties and 7 annotation properties). The full ontology statistics of ICEO are accessible on the Ontobee^25^ ICEO statistics page (http://www.ontobee.org/ontostat/ICEO).

**Table 1:** Summary of ontology terms in ICEO as of January 23, 2021.

Ontology Names	Classes	Object properties	Annotation properties	Instance	Total
ICEO (Integrative and conjugative element ontology)	4128	11	21	0	4160
BFO (Basic Formal Ontology)	19	11	2	0	32
OGG (Ontology of Genes and Genomes)	26448	1	8	0	26457
NCBITaxon (NCBI organismal classification)	321	0	0	0	321
GO (Gene Ontology)	11	0	0	0	11
RO (Relation Ontology)	1	27	7	1	36
Other ontologies*	15	30	49	6	100
**Total**	30942	53	80	6	31081

Note: *the name and statistics of other ontologies used in ICEO can be found on the Ontobee website: http://www.ontobee.org/ontostat/ICEO.

### ICEO applications

**Fig. 3:**
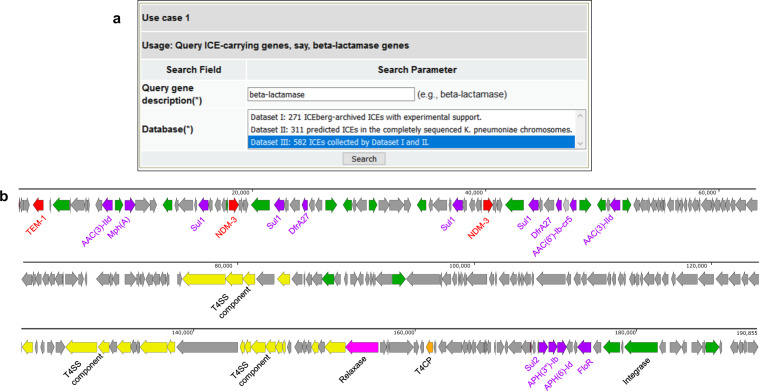
The SPARQL query of all beta-lactamase genes over ICEO. **(a)** The ICEberg SPARQL query interface of use case 1 (https://bioinfo-mml.sjtu.edu.cn/ICEberg2/search_sparql.php). In this case, the user could input any keywords of gene description and select the targeted RDF database. The result web page is shown in Supplementary Figure S6. The source SPARQL query code of this example is provided in the Supplementary Materials. **(b)** The genetic structure of ICE*Kpn*QD23-1. ICE*Kpn*QD23-1 has 52% GC content, lower than the average GC content of the chromosomes of *K. pneumoniae* QD23 is 57%. Red, beta-lactamase genes. Purple, the other antibiotic resistance genes. Green, integrase or transposase genes. Yellow, genes coding for T4SS components.

Formatted in the machine-understandable OWL format, ICEO can be used for various applications, such as SPARQL query (Fig. 3) and DL query (Supplementary Figure S5). SPARQL query is designed to query an RDF (Resource Description Framework) triple store, and handle complex joins and relationships of ICEO.

#### Use Case 1: Query ICE-carrying beta-lactamase genes over ICEO

The beta-lactam resistance genes, especially carbapenemase genes, are frequently carried by the plasmids of Gram-negative bacteria. One important question to ask is whether these beta-lactamase genes are horizontally transferred into the chromosome backbones. ICEO has already collected, integrated, and defined all the gene information of ICEs and the relationship with other ICE-related terms in the RDF store. As shown in Fig. 3a, with a SPARQL query for ‘beta-lactamase’ over ICEO, we can find beta-lactamase genes in 4 ICEs with experimental supports in 4 species and 2 putative ICEs of *K. pneumoniae* (Supplementary Figure S6a). For example, the self-transferable ICE*clc*-like ICE*Pae*690 (ICEO ID: ICEO_0000111) in *Pseudomonas aeruginosa* carries a *bla*_GES-6_ gene^26^, coding for a class A beta-lactamase with carbapenemase activity (ICEO ID: OGG_3_1242632754). Notably, the 190-kb ICE*Kpn*QD23-1 from *K. pneumoniae* QD23 (GenBank accession: CP042858) has two metallo-beta-lactamase NDM-3 genes and one broad-spectrum beta-lactamase TEM-1 gene, which indicates the organization of antibiotic resistance island. Further investigation shows that ICE*Kpn*QD23-1 has a total of 17 ARGs and a high number of insertion sequences (IS). The detailed genetic structure of ICE*Kpn*QD23-1 is shown in Fig. 3b. The BLASTn search of the *bla*_NDM-3_-carrying ICE*Kpn*QD23-1 against the NCBI *nr* database shows high sequence similarity to *Escherichia coli* CRE1540 plasmid p1540-2 (GenBank accession: CP019053)^27^, indicating that ICE*Kpn*QD23-1 might be a ‘chromosomal insertion’ version of p1540-2 (Supplementary Figure S6b). This result showed that using the SPARQL query to perform similar tasks is an efficient choice.

#### Use Case 2: Query a specific type of ICE genes, like non-ribosomal peptide synthetase genes

**Fig. 4:**
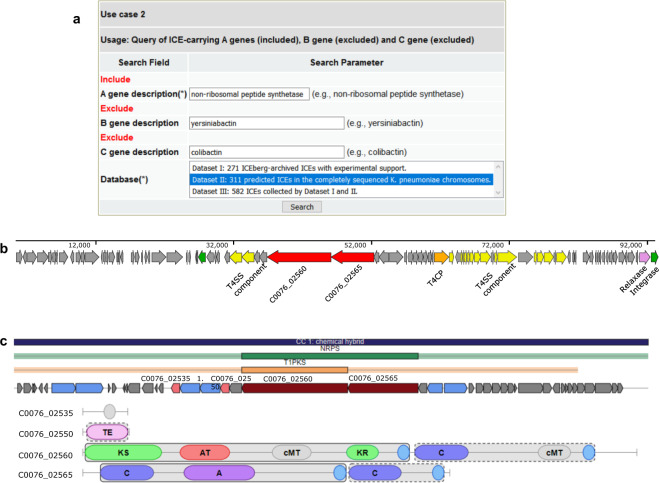
The genetic structure of ICE*Kpn*LS357-1 and the domain composition of nonribosomal peptide synthetase - polyketide synthase (NRPS-PKS) hybrids. **(a)** The ICEberg SPARQL query interface of use case 2 (https://bioinfo-mml.sjtu.edu.cn/ICEberg2/search_sparql.php). The result web page is shown in Supplementary Figure S7. The source SPARQL query code of this example is provided in the Supplementary Materials. **(b)** Schematic representation of ICE*Kpn*LS357-1. ICE*Kpn*LS357-1 has 45% GC content compared to 57% for the chromosomes of *K. pneumoniae* LS357. The functional module related genes are annotated with different colors and labels below. Two red arrows highlight the genes coding NRPS and Type I PKS. The remaining ORFs of the ICE are represented by gray arrows and arrowheads. **(c)** Domain annotation of NRPS and PKS by using antiSMASH.

The non-ribosomal peptide synthetase (NRPS)-associated biosynthesis of two siderophores, yersiniabactin and colibactin, have been characterized within the ICE*Kp1* of *K. pneumoniae*. Another example is to explore the ICE-encoding other NRPS by the query of the ICEO gene description that includes ‘non-ribosomal peptide synthetase’ but neither ‘yersiniabactin’ nor ‘colibactin’ (Fig. 4a, https://bioinfo-mml.sjtu.edu.cn/ICEberg2/search_sparql.php, SPARQL Query over ICEO Use case 2 There are 215 NRPS genes found among 113 ICEs of 109 *K. pneumoniae*. Interestingly, 6 NRPS genes are only annotated as “non-ribosomal peptide synthetase”, indicating that they might be not related to the biosynthesis of yersiniabactin or colibactin (Supplementary Figure S7**)**. Further investigation quickly leads us to a 92-kb ICE*Kpn*LS357-1 of *K. pneumoniae* LS357 (CP025639) **(**Fig. 4b**)**. It encodes NRPS-PKS hybrid that is found in only 6 *K. pneumoniae* strains and is not present within the antiSMASH database^28^, suggesting it might code for an unknown compound **(**Fig. 4c**)**. The LS357 strain was assigned to ST23 and was isolated from liver abscess puncture fluid in China, and more experiments are needed to investigate whether this compound is a new virulence factor.

## Discussion

In this study, we developed the ICEO that ontologically represents the complex hierarchical structure of ICEs, ICE components, and the relations among ICEs and ICE components. The ICEO representation of the experimentally verified ICE knowledge supports computer-assisted data integration, efficient query, and reasoning.

Now ICEO is built by standardizing and integrating the rich information from the ICEberg database^6^. While the data stored in the ICEberg database are well structured for querying and processing in the database system, the data are not easily understood by machines outside the database, the ICEO ontology is machine-interpretable, portable, and integrable so that is more suitable for data sharing and cross-data analysis^29^. Our two use cases by using SPARQL query also demonstrate that ICEO can help perform tasks that are difficult to be done in the current ICEberg database. Besides, with DL query over ICEO, Supplementary Figure S5 shows that we were able to easily query the virulence factors for any level of ICEs. Currently, ICEberg will label “VF” for those ICEs that are virulence factors. However, it is still impossible for users to query all VFs for a specific bacterial group. Rather than being only a translation of the ICEberg database, ICEO and ICEO-based features are being explored to be integrated into ICEberg (Figs. 3a,  4a, and Supplementary Figure S8). Furthermore, ICEO might facilitate ontology-based ICE literature mining as shown in many other ontology-based research domains^30,31^.

ICEO is the first BFO-based ICE ontology. Toussaint *et al*. developed the MeGO, a Gene Ontology dedicated to the functions of mobile genetic elements, and used it in the ACLAME database (A CLAssification of Mobile genetic Elements)^32–34^. MeGO is a non-OBO ontology expanded from the Phage Ontology (PhiGO). It contains 375 classes, a single object property (which is “*part of*”), and 22 annotation properties. Most of the MeGO terms are related to phages, GO, and sequences. Only a few terms directly related to ICEs are included in MeGO. MeGO does not include any specific ICEs and ICE gene components. It is also noted that the MeGO ontology and ACLAME database have not been updated in the past six years. In comparison, ICEO is systematically developed by aligning with the widely used BFO upper-level ontology and following the OBO Foundry principles. ICEO represents the complicated gene components, functional modules, and related information about T4SS-type ICEs and AICEs, making it possible to perform the automatically computer-assisted reasoning, query, and advanced analysis of ICE data.

ICEO will be further developed in the future. We will represent more known information about T4SS-type ICEs. T4SS-type ICEs broadly exist in Gram-negative and Gram-positive bacteria. However, due to the differences in cell membrane structure, Gram-positive and Gram-negative bacteria have different T4SS organization and features and are associated with different T4SS-type ICEs. Such differential characteristics will be further categorized, modeled, and represented in ICEO. Besides, only 11 experimentally verified actinomycete ICEs (AICEs) are included in ICEO. Compared with T4SS-type ICEs, AICEs are less commonly seen in bacteria and only found in *Actinobacteria*. However, AICEs are important in developing useful tools for the genetic engineering of *Actinobacteria*^35^. In the future, we plan to represent and analyze AICE information in ICEO more systematically.

## Materials and Methods

### ICE data source

The information of 271 experimentally verified ICEs data was retrieved from the ICEberg database. Besides, 505 completely sequenced chromosomes of *K. pneumoniae* were downloaded from the NCBI RefSeq database in March 2020 and then the putative ICEs in these chromosomes were identified by ICEfinder^6^. After manual curation, a total of 311 putative ICEs were obtained.

### ICEO ontology development strategy

The development of ICEO follows the Open Biological and Biomedical Ontologies (OBO) Foundry principles^13^, such as openness, collaboration, use of a common shared syntax. To support the data FAIRness (Findable, Accessible, Interoperable, and Reusable)^36^, the eXtensible Ontology Development (XOD) strategy^19^ was also applied for the ontology development of ICEO. The XOD strategy recommends the reuse of existing terms and semantic relations from reliable ontologies, development and application of well-established ontology design patterns (ODPs), and involvement of community efforts for new ontology development^19^. The application of the XOD strategy made ICEO effectively integrated with other ontologies in the OBO library. The release of ICEO followed the MIRO (Minimum Information for Reporting of an Ontology) guidelines^37^ to ensure to meet the standard requirements of ontologies publication.

### ICE-related ontology term reuse

To support ontology interoperability and avoid reinventing the wheel, related existing terms from reliable ontologies were imported into ICEO via an Ontofox import strategy^38^. The external ontologies used here include Ontology of Genes and Genomes (OGG)^21^, Gene Ontology (GO)^18^, Relation Ontology (RO)^23^, and a taxonomy ontology of NCBI organismal classification (NCBITaxon)^22^.

### OGG-related gene term generation and usage

The original OGG gene ID and gene label assignments^21^ need to be modified. To avoid gene ID conflicts, the original OGG designed a special scheme to automatically assign gene IDs by mapping ontology ID with NCBITaxon IDs and NCBI Gene IDs^21^. However, Gene IDs are no longer provided and used by NCBI for the sequence records in non-reference strains^39^. Meanwhile, NCBITaxon IDs do not exist for many ICE-containing organisms such as *Escherichia coli* strain ECOR31. OGG also faces the challenge of avoiding gene label redundancy. OGG usually uses gene name or locus tag as the gene label, while many genes in different organisms have the same names in NCBI GenBank records.

By working with the OGG development group^21^, we developed an OGG extension strategy of generating new OGG IDs for gene assignments for ICE-related genes. Simply put, this strategy assigns OGG gene IDs using NCBI “locus_tag” identifiers commonly seen in GenBank gene records. For the gene label, if a gene name is available for a gene, the gene label will be assigned as ‘locus_tag(gene_name)’; if not, the gene label will be ‘locus_tag’. Such a naming strategy allows us to develop computer programs to automatically generate readable and nonredundant ICEO gene labels.

### New ICEO term generation

Compared to manually adding terms and logical definitions, pattern-based ontology development strategies^40,41^ are faultless and much more efficient. Here, a general ontology design pattern (ODP) was developed to logically link different components related to ICEs. Based on this ODP, we used the web-based Ontorat program^40^ to automatically add new ontology terms, hierarchies, annotations, and logical relations between entities. The Protégé-OWL editor (version 5.2)^42^ was used for manual ICEO processing, visualization, and editing. ICEO-specific terms were generated by assigning new ICEO identifiers with the prefix “ICEO_” followed by auto-generated 7 digits. The Hermit reasoner (http://hermit-reasoner.com/) was applied for semantic consistency checking and inferencing. Deprecated classes, including entities that become removed, split, or redefined, will be labeled as obsolete with *“owl:deprecated”* annotation property.

### ICEO knowledge query and analysis

The knowledge stored in the ICEO ontology can be queried through different approaches. In this study, we used the Description Logic (DL) query and SPARQL query. Description logic is a formal knowledge representation language to describe a given domain by defining relevant concepts and asserting properties of individuals (also called axioms)^43^. After reasoning the represented concepts and axioms, the DL query can infer hidden knowledge. Since OWL (the format of ICEO) is based on DLs, the DL query on ICEO can be easily performed in a DL Query platform such as one inside the Protégé OWL editor^42^. As a standard query language recommended by World Wide Web Consortium (W3C), SPARQL is a recursive acronym for SPARQL Protocol and RDF Query Language (https://www.w3.org/TR/sparql11-query/). SPARQL supports efficient query, retrieval, and manipulation of data stored in graph-based RDF data stores. For the ICEO-specific SPARQL query, ICEO was stored in an Open Link Virtuoso database system (RDF store). An ICEberg integrated SPARQL query interface (https://bioinfo-mml.sjtu.edu.cn/ICEberg2/search_sparql.php) with some useful query models was designed and developed using JavaScript, PHP, and HTML. Users can implement some commonly used SPARQL queries toward ICEO by selecting or inputting keywords we designed without the need of learning and writing SPARQL query scripts. And more query models will be added under the users’ requests. As an OBO library ontology (http://www.obofoundry.org/ontology/iceo.html), ICEO was automatically updated and stored in Ontobee RDF triple store^25^. Users with good SPARQL query experience could also construct custom SPARQL queries using the Ontobee SPARQL query interface (http://www.ontobee.org/sparql).

## Supplementary information

Supplementary information

## Data Availability

The data and materials introduced are all openly available at figshare (10.6084/m9.figshare.17008543.v2)^44^. The latest version of ICEO is accessible for visualization and downloading from the Ontobee ontology repository website: http://www.ontobee.org/ontology/ICEO, NCBO’s BioPortal website: https://bioportal.bioontology.org/ontologies/ICEO, or OLS (Ontology Lookup Service) website: https://www.ebi.ac.uk/ols/ontologies/iceo.

## Code availability

The source code of ICEO can be downloaded from the ICEO GitHub website: https://github.com/ontoice/ICEO. Term requests and updating can also be reached on the ICEO GitHub issues tracker (https://github.com/ontoice/ICEO/issues). And wide community participation in ICEO development and applications is highly welcome.
