# Supplementary material for: ICEO, a biological ontology for representing and analyzing bacterial integrative and conjugative elements
Source: Sci Data. 2022 Jan 20;9:11. doi: 10.1038/s41597-021-01112-5 (PMC8776819; doi:10.1038/s41597-021-01112-5)
Supplement: Supplementary file 1 — Supplementary information [file 41597_2021_1112_MOESM1_ESM.pdf]

ICEO, a biological ontology for representing and analyzing bacterial integrative and conjugative elements

Supplementary materials

Figure S1. Demonstration of the naming strategy of gene IDs and gene labels in ICEO.

Figure S2. Demonstration of ICEO linkages of different entities and representation of the virulence factor gene *ybtE* of the accessory module in ICE*Kp1.*

Figure S3. Demonstration of ICEO linkages of different entities and representation of the antibiotic resistance gene *strB* of the accessory module in SXT(MO10).

Figure S4. Demonstration of ICEO linkages of different entities and representation of the relaxase gene *tecA* of the conjugative module in Tn*916.*

Figure S5. DL query of all virulence factor genes of ICE*Kp1* over ICEO.

Figure S6. The SPARQL query of all beta-lactamase genes over ICEO.

Figure S7. Part screenshot of the result web page of use case 2

Figure S8. The integration between ICEO ontology and ICEberg database using ICE*Kp1* as an example.

Attached **SPARQL query source codes**, tested on the Virtuoso SPARQL Query Editor.

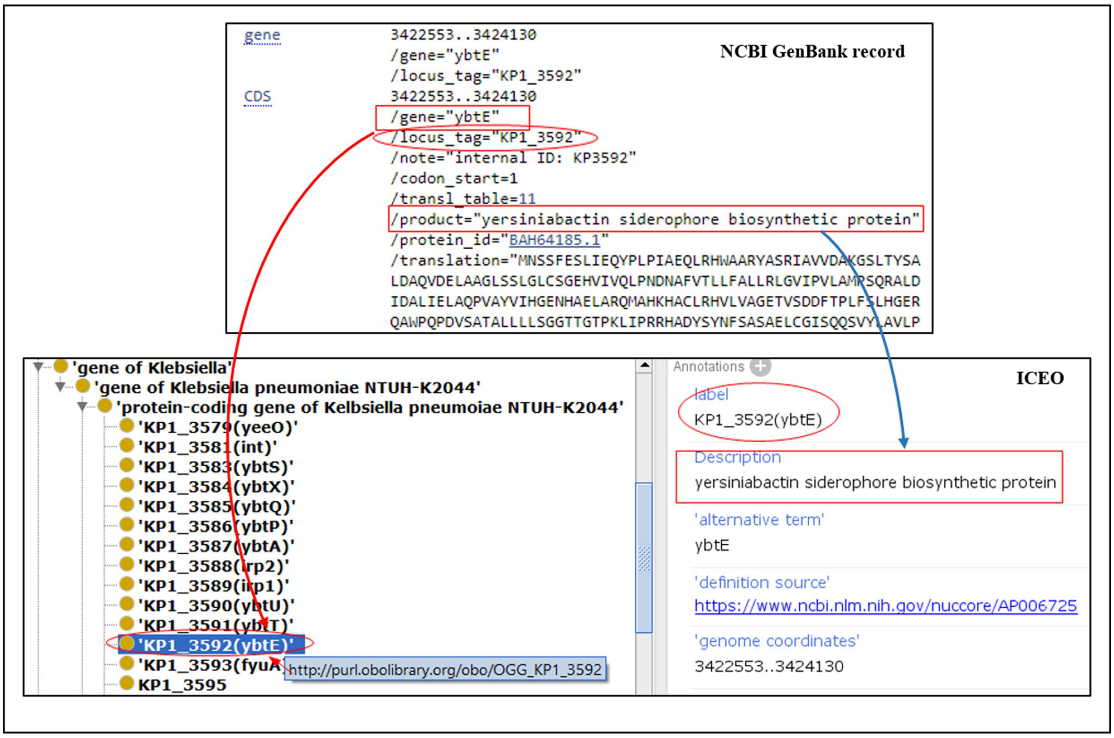

Figure S1. Demonstration of the naming strategy of gene IDs and gene labels in ICEO. In this example, the gene *ybtE* that is archived in the NCBI GenBank database is assigned with gene label ‘KP1_3592(ybtE)’ in ICEO, and its corresponding gene ID is “OGG_KP1_3592”. See the detailed explanation in the main text.

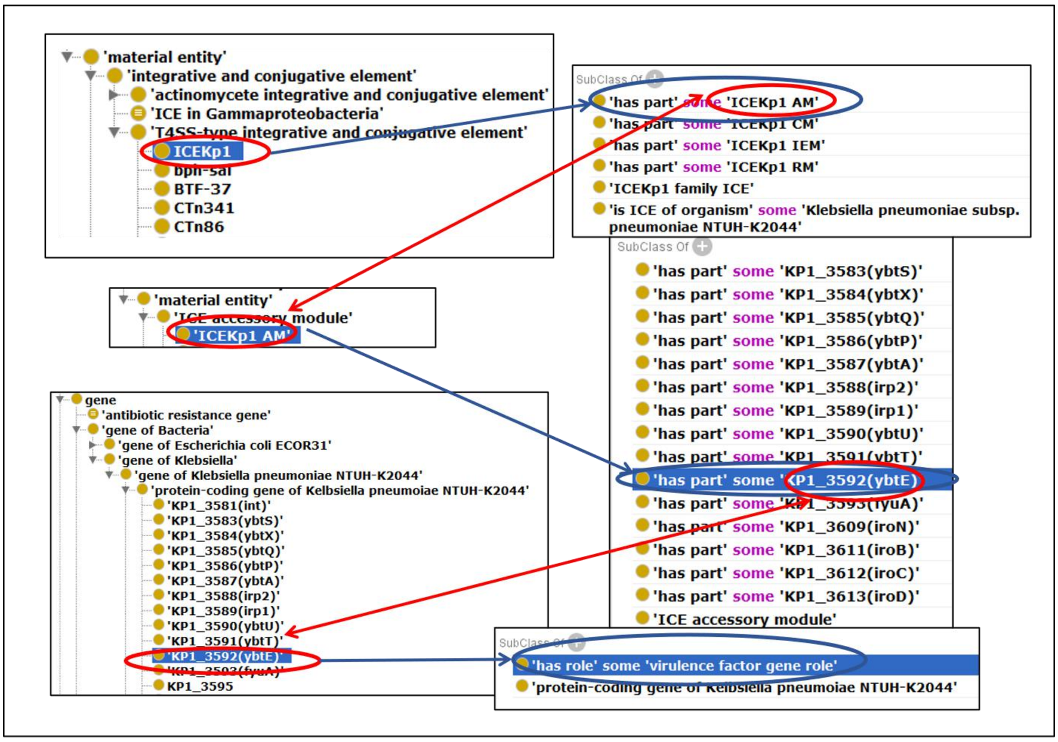

Figure S2. Demonstration of ICEO linkages of different entities and representation of the virulence factor gene *ybtE* of the accessory module in ICE*Kp1*. ICE*Kp1* '*has part*' an accessory module 'ICE*Kp1* AM'. This module '*has part*' a *ybtE* gene, which '*has role*' a virulence factor gene role.

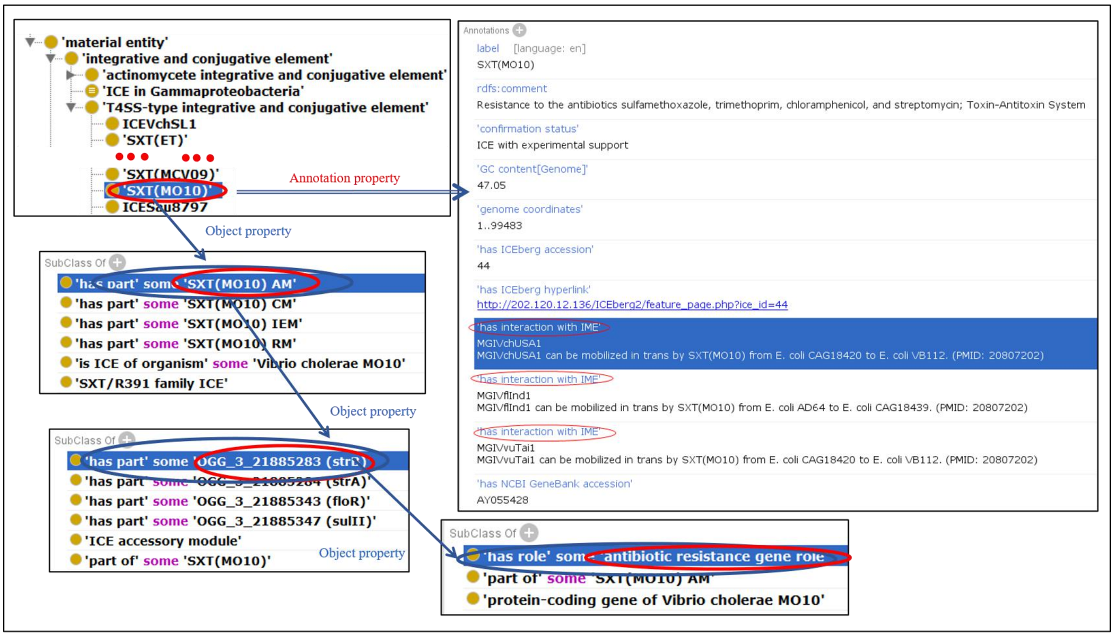

Figure S3. Demonstration of ICEO linkages of different entities and representation of the antibiotic resistance gene *strB* of the accessory module in SXT(MO10). SXT(MO10) '*has part*' an accessory module 'SXT(MO10) AM'. This module *'has part*' an *strB* gene, which '*has role*' an antibiotic resistance gene role.

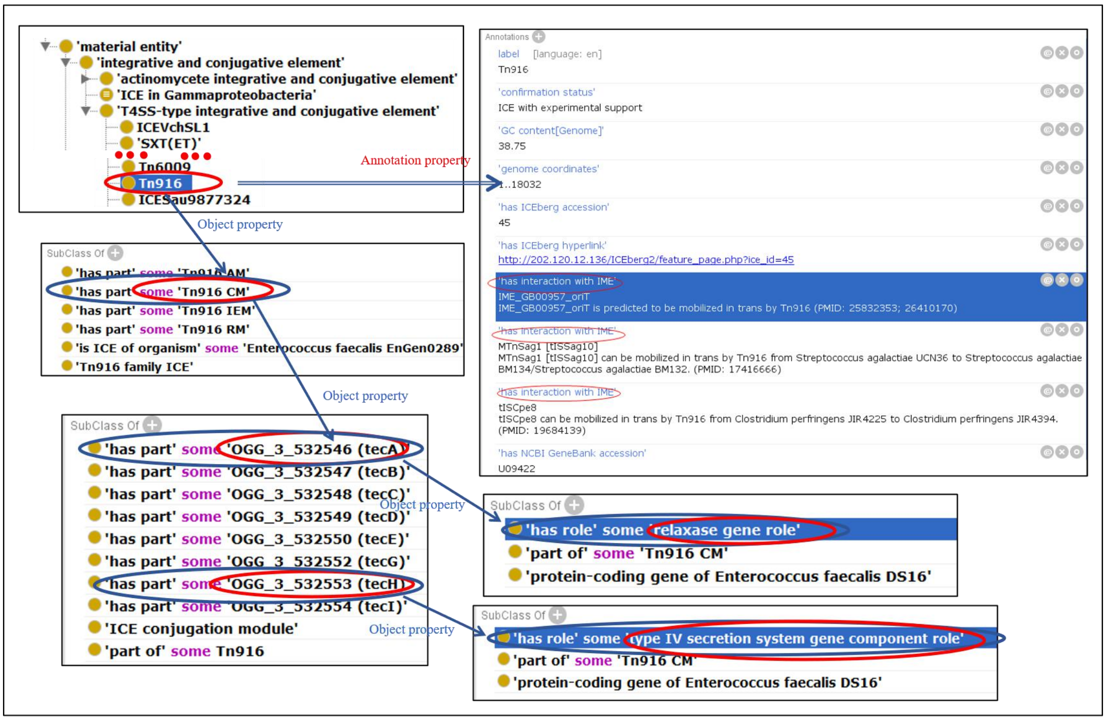

Figure S4. Demonstration of ICEO linkages of different entities and representation of the relaxase gene *tecA* of the conjugative module in Tn*916*. Tn*916* '*has part*' an accessory module 'Tn*916* CM'. This module '*has part*' a *tecA* gene, which '*has role*' a relaxase gene role.

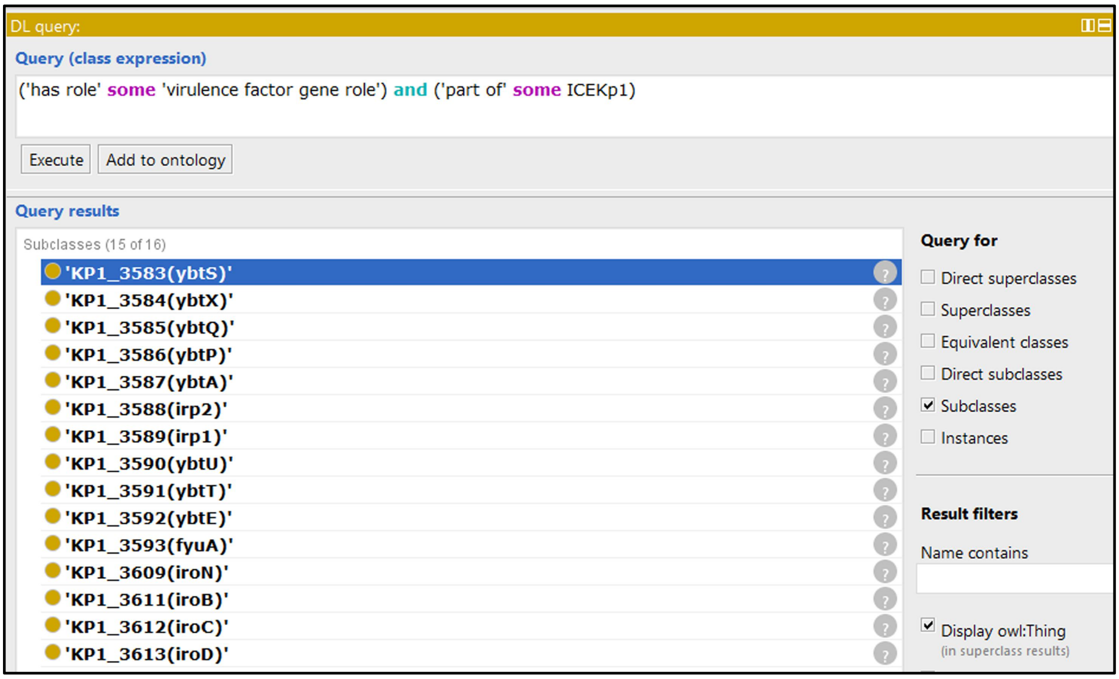

Figure S5. DL query of all virulence factor genes of ICE*Kp1* over ICEO.

The DL Query was performed using the Protégé 5.2 OWL editor. The query code is shown on the top, and the query results are displayed at the bottom. A total of 15 results were identified.

An example competency question often asked by users is: *What genes in the ICEKp1 encode virulence factors?*

In ICEO, a virulence factor gene is logically defined as “something that *has role* some *virulence factor gene role*”. To answer this question, the following query was simply performed using a DL Query function:

('has role' some 'virulence factor gene role') and ('part of' some ICEKp1)

Our query easily identified 15 genes that are part of the specific ICE*Kp1* and also encode for virulence factors in the host bacterium of the ICE*Kp1*. Consistent with the literature [11,24], ICE*Kp1* is responsible for transporting these virulence factor genes to *K. pneumoniae* strain NTUH-K2044, and such virulence factor gene transportation is indeed critical to make the bacterium virulent.

a

callret-1	callret-3	gene_description	callret-6
ICE-GI1	Bpet1117	"metallo-beta-lactamase family protein"	Bordetella petrii DSM 12804
ICEPae690	OGG_3_1242632754	"Carbapenem-hydrolyzing class A beta-lactamase GES-6"	Pseudomonas aeruginosa FFUP_PS_690
ICEPmu1	Pmu_03440(blaOXA-2)	"beta-lactamase OXA-2 protein"	Pasteurella multocida 36950
ICEMcSym(1271)-alpha	Mesci_5712	"beta-lactamase"	Mesorhizobium ciceri biovar biserrulae WSM1271
ICEKpnINF249-2	CTI54_RS26675	"class A broad-spectrum beta-lactamase TEM-1"	Klebsiella pneumoniae INF249
ICEKpnINF249-2	CTI54_RS26695	"class A extended-spectrum beta-lactamase CTX-M-15"	Klebsiella pneumoniae INF249
ICEKpnQD23-1	FU841_RS24655	"subclass B1 metallo-beta-lactamase NDM-3"	Klebsiella pneumoniae QD23
ICEKpnQD23-1	FU841_RS24545	"class A broad-spectrum beta-lactamase TEM-1"	Klebsiella pneumoniae QD23
ICEKpnQD23-1	FU841_RS24790	"subclass B1 metallo-beta-lactamase NDM-3"	Klebsiella pneumoniae QD23

b

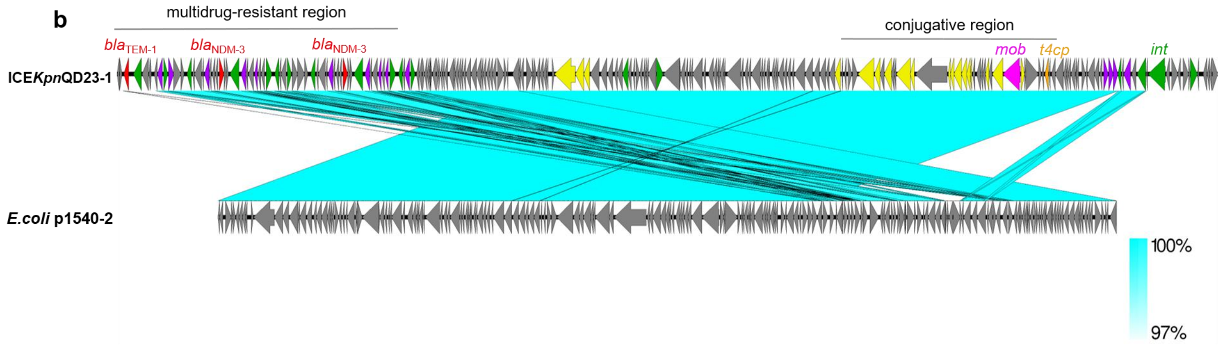

Figure S6. The SPARQL query of all beta-lactamase genes over ICEO (use case 1, Figure 3)**. (a)** Part screenshot of the result web page shows the list of the related ICE, locus tag, gene description, and the host bacterium of ICE. Users can also get more information about these ICEs by clicking the corresponding ICEberg link and/or ICEO term URI on the result web page. **(b)** Synteny between the 190-kb ICE*Kpn*QD23-1 of *Klebsiella pneumoniae* QD23 and the 155-kb plasmid p1540-2 of *Escherichia coli* CRE1540. The figure was constructed using EasyFig [44]. Red, beta-lactamase genes. Purple, the other antibiotic resistance genes. Green, integrase or transposase genes. Yellow, genes coding for T4SS components.

callret-1	callret-3	gene_description	callret-6
ICEKpnTGH8-1	AOD72_RS04200	"non-ribosomal peptide synthetase"	Klebsiella pneumoniae subsp. pneumoniae TGH8
ICEKpnCR14-2	A6P37_RS09710	"non-ribosomal peptide synthetase"	Klebsiella pneumoniae CR14
ICEKpnTVGHCRE225-1	C0233_RS05360	"non-ribosomal peptide synthetase"	Klebsiella pneumoniae TVGHCRE225
ICEKpnKP9-1	CWT06_RS00885	"non-ribosomal peptide synthetase"	Klebsiella pneumoniae KP9
ICEKpnLS357-1	C0076_RS02565	"non-ribosomal peptide synthetase"	Klebsiella pneumoniae LS357
ICEKpn16_GR_13-1	A6D82_RS02220	"non-ribosomal peptide synthetase"	Klebsiella pneumoniae 16_GR_13

Figure S7. Part screenshot of the result web page of use case 2 (Figure 4). The columns from left to right are the NRPS gene-containing ICE, the locus tag, gene description, and the host bacterium of ICE. Users can also get more information about these ICEs by clicking the corresponding ICEberg link and/or ICEO term URI on the result web page.

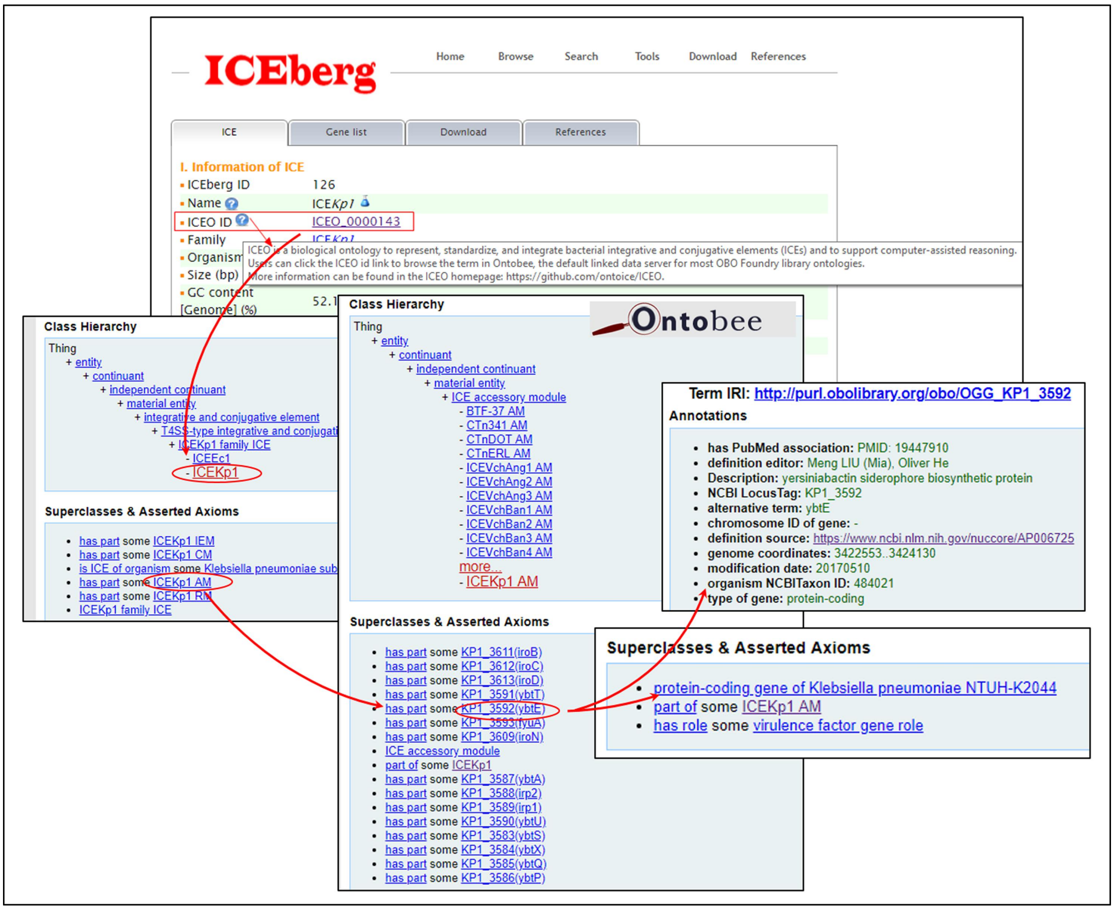

Figure S8. The integration between ICEO ontology and ICEberg database using ICE*Kp1* as an example.

ICEO_0000143, the ICEO ID for the specific ICE ICE*Kp1* in *K. pneumoniae* strain NTUH-K2044, is now available on the ICEberg website. A click of the ICEO ID on the ICEberg page allows a user to access the Ontobee website and identify more detailed information about the ICE. In addition to its class hierarchy, we can also find asserted axioms. For example, ICE*Kp1* has part of an accessory module 'ICE*Kp1* AM', which has many gene components such as 'KP1_3592(ybtE)'. Here the *ybtE* is the gene symbol and the KP1_3592 is the locus tag of the gene. In this example, ICEO ID is listed for the standardization of ICE*Kp1*. With a click on the ICEO ID "ICEO_000143" inside the ICEberg ICE*Kp1* data page, users can browse the ontological hierarchy, modular relation, and recursive information of the ICE*Kp1*. The linkage of the ICEberg item to the ICEO contents makes the seamless interaction between these two resources.

Attached SPARQL query source codes, tested on the Virtuoso SPARQL Query Editor

(http://sparql.hegroup.org/sparql/**)**

Use case 1:

```
## To find all the beta-lactamase genes carried by ICEs
PREFIX is_ICE_of_organism: <http://purl.obolibrary.org/obo/ICEO_0000020>
PREFIX is_gene_of_ICE: <http://purl.obolibrary.org/obo/ICEO_0000051>
PREFIX description: <http://purl.org/dc/elements/1.1/description>
PREFIX has_part: <http://purl.obolibrary.org/obo/BFO_0000051>
PREFIX has_ICEberg_hyperlink: <http://purl.obolibrary.org/obo/ICEO_0000063>

SELECT DISTINCT IRI(?iceberg_link) STR(?ice_label) ?ice
STR(?gene_label) ?gene ?gene_description STR(?organ_label) ?organ
from <http://purl.obolibrary.org/obo/merged/ICEO>
WHERE {
?ice rdfs:label ?ice_label .
?ice has_ICEberg_hyperlink: ?iceberg_link .
?ice rdfs:subClassOf ?ice_restriction .
?ice_restriction owl:onProperty is_ICE_of_organism:;
owl:someValuesFrom ?organ .
?organ rdfs:label ?organ_label .
?gene rdfs:label ?gene_label .
?gene rdfs:subClassOf ?gene_restriction .
?gene_restriction owl:onProperty is_gene_of_ICE:; owl:someValuesFrom ?ice .
?gene description: ?gene_description .
FILTER regex(?gene_description, 'beta-lactamase', 'i') .
}
```

Use case 2:

```
# to find all the non-ribosomal peptide synthetase (NRPS) genes by ICEs, excluding genes coding for yersiniabactin and colibactin
PREFIX is_ICE_of_organism: <http://purl.obolibrary.org/obo/ICEO_0000020>
PREFIX is_gene_of_ICE: <http://purl.obolibrary.org/obo/ICEO_0000051>
PREFIX description: <http://purl.org/dc/elements/1.1/description>
PREFIX has_ICEberg_hyperlink: <http://purl.obolibrary.org/obo/ICEO_0000063>
PREFIX confirmation_status: <http://purl.obolibrary.org/obo/ICEO_0000340>

SELECT DISTINCT IRI(?iceberg_link) STR(?ice_label) ?ice
STR(?gene_label) ?gene ?gene_description STR(?organ_label) ?organ
from <http://purl.obolibrary.org/obo/merged/ICEO>
WHERE {
?ice rdfs:label ?ice_label .
?ice has_ICEberg_hyperlink: ?iceberg_link .
?ice rdfs:subClassOf ?ice_restriction .
?ice_restriction owl:onProperty is_ICE_of_organism:;
owl:someValuesFrom ?organ .
?ice confirmation_status: "putative ICE predicted with ICEfinder".
?organ rdfs:label ?organ_label .
?gene rdfs:label ?gene_label .
?gene rdfs:subClassOf ?gene_restriction .
?gene_restriction owl:onProperty is_gene_of_ICE:; owl:someValuesFrom ?ice .
?gene description: ?gene_description .
FILTER regex(?gene_description, 'non-ribosomal peptide synthetase', 'i') .
FILTER (!regex(?gene_description, 'yersiniabactin', 'i')) .
FILTER (!regex(?gene_description, 'colibactin', 'i')) .
}
```
